# Operational integration in primary health care: patient encounters and workflows

**DOI:** 10.1186/s12913-017-2702-5

**Published:** 2017-11-29

**Authors:** Dimitra Sifaki-Pistolla, Vasiliki-Eirini Chatzea, Adelais Markaki, Kyriakos Kritikos, Elena Petelos, Christos Lionis

**Affiliations:** 1 Clinic of Social and Family Medicine, School of Medicine, University of Crete, University Campus, Voutes, P.O. Box 2208, Heraklion, 71003 Crete, Crete Greece; 2 School of Nursing, University of Alabama at Birmingham, Birmingham, USA; 3 Institute of Computer Science, FORTH, Vassilika Vouton, 70013 Crete, Greece

**Keywords:** Primary health care, Operational integration, Integrated care, Clinical pathways, Structure, Outcome, Process assessment (healthcare), Quality indicators (healthcare), Greece

## Abstract

**Background:**

Despite several countrywide attempts to strengthen and standardise the primary healthcare (PHC) system, Greece is still lacking a sustainable, policy-based model of integrated services. The aim of our study was to identify operational integration levels through existing patient care pathways and to recommend an alternative PHC model for optimum integration.

**Methods:**

The study was part of a large state-funded project, which included 22 randomly selected PHC units located across two health regions of Greece. Dimensions of operational integration in PHC were selected based on the work of Kringos and colleagues. A five-point Likert-type scale, coupled with an algorithm, was used to capture and transform theoretical framework features into measurable attributes. PHC services were grouped under the main categories of chronic care, urgent/acute care, preventive care, and home care. A web-based platform was used to assess patient pathways, evaluate integration levels and propose improvement actions. Analysis relied on a comparison of actual pathways versus optimal, the latter ones having been identified through literature review.

**Results:**

Overall integration varied among units. The majority (57%) of units corresponded to a basic level. Integration by type of PHC service ranged as follows: basic (86%) or poor (14%) for chronic care units, poor (78%) or basic (22%) for urgent/acute care units, basic (50%) for preventive care units, and partial or basic (50%) for home care units. The actual pathways across all four categories of PHC services differed from those captured in the optimum integration model. Certain similarities were observed in the operational flows between chronic care management and urgent/acute care management. Such similarities were present at the highest level of abstraction, but also in common steps along the operational flows.

**Conclusions:**

Existing patient care pathways were mapped and analysed, and recommendations for an optimum integration PHC model were made. The developed web platform, based on a strong theoretical framework, can serve as a robust integration evaluation tool. This could be a first step towards restructuring and improving PHC services within a financially restrained environment.

**Electronic supplementary material:**

The online version of this article (10.1186/s12913-017-2702-5) contains supplementary material, which is available to authorized users.

## Background

The concept of integration has received a lot of attention in the literature, although its definition and scope varies across settings [1–3]. The World Health Organization (WHO) has defined integrated care delivery as *“[…] the management and delivery of health services so that clients receive a continuum of preventive and curative services, according to their needs over time and across different levels of the health system”* [1]. Literature supports the premise that integration results in better health outcomes and minimises overall healthcare costs [2, 3]. Main benefits include patient orientation, equity, quality, accessibility, efficiency, continuity of care, and cost-effectiveness.

The first integrated care models were introduced during the 1980s in the USA [4]. Those models focused on chronic disease and care provision according to patient needs [5] and significantly influenced developments in other countries. The Netherlands and the United Kingdom (UK), both countries with strong primary healthcare (PHC) systems and gatekeeping, have adopted integrated approaches to link health promotion and disease prevention to disease management and self-management support [6]. Electronic prescribing, integration of pharmacies within healthcare units, comprehensive training of healthcare professionals (HCPs), use of community resources, and an accessible referral system with optimised patient flows in multidisciplinary centres, have all contributed towards reduced healthcare costs and more efficient reallocation of resources [7, 8]. In 2006, the last wave of healthcare reforms in the Netherlands focused on sustaining the successful innovations of previous decades. Strong emphasis was given on improving information technology (IT) services, coordinated and comprehensive chronic care and optimum utilisation of community resources [7]. In the UK, the “Quality and Outcomes Framework (QOF)” considered as best practice the adoption of electronic records and other measurable variables that facilitate quality monitoring and benchmarking data [8]. These best practices are crucial in ensuring sound resource allocation, especially in countries with highly burdened healthcare systems and less developed PHC, such as Greece [9]. Therefore, such experiences could guide countries with fragmented systems towards effective reforms in optimising unit and patient-level integration and introducing standardised processes. An added benefit could be the provision of valuable information for evidence-based policy in terms of allocating or reallocating resources at system level.

Having been subjected to a harsh austerity period, Southern European countries share similar healthcare system characteristics and challenges in PHC service delivery [9]. In 2000 and 2008, Greece and Spain exhibited a rapid expansion of public spending, while in Italy and Portugal the trend was moderate [9–11]. Despite restricted coverage of PHC services, Italy has achieved a high degree of system integration and an effective way of managing public funding and private healthcare expenditure [9]. Nevertheless, the lack of standardised processes and protocols for patient pathways, as well as for addressing the needs of patients with multiple morbidities, represents an important commonality for all these systems.

During the last thirty years, Greece has attempted to strengthen its national health system (NHS), by expanding and standardising PHC, initially in rural, and more recently in urban areas. Despite efforts and an intense debate lasting more than 15 years, Greece is still lacking a sustainable, evidence-based integrated model. As a result, integration still remains a largely neglected issue in the country’s health policy agenda [12, 13]. The existing socio-economic hardships inflicted by the prolonged financial austerity, as well as the recent refugee and migrant crisis, render the need for healthcare reform urgent. Moreover, the country’s rapidly aging population, along with the high incidence of mental health disorders [14] and the growing burden of chronic diseases, [15] necessitate immediate actions towards an integrated, multidisciplinary network of well-coordinated and cost-effective services. Lack of integration can result in fragmentation of care and poor health outcomes, [16] as well as in problems related to funding, planning, effectiveness and operation of the healthcare system[17]. Substantial healthcare budget cuts and prolonged delay of major reform are jeopardising the NHS, putting it at risk of becoming unsustainable and ultimately, obsolete [18]. Failing to achieve immediate policy and structural changes to this direction, could increase the risk of potential NHS collapse with numerous adverse consequences [13]. Thus, it is vital to develop and implement policy well-aligned to a strategic vision towards integrated PHC. This can be a challenging and arduous process, considering that it requires major NHS reform along with changes in organisational culture [12].

Having identified the above urgent need, the Clinic of Social and Family Medicine (CSFM) (School of Medicine, University of Crete) conducted a large nationally funded research project with a two-fold aim: 1) to assess the level of operational integration within PHC units by utilising standardised quality processes which included mapping and evaluation of both unit-level and patient-level integration and 2) to develop an optimum model of operational integration tailored to the Greek PHC system. This paper aims to present integration findings regarding existing patient care pathways and to suggest an alternative pathway model for optimum integration.

## Methods

The project was funded by the Greek National Strategic Reference Framework (NSFR) 2007–2013 and was conducted from June 2012 to November 2015. The Health Region of Crete (Ref. #9674), the Health Region of the Aegean Islands (Ref. #1136) and the Ministry of Health and Solidarity (Ref. #38865) all granted ethics approval for the conduct of this study.

### Theoretical framework

Primary health care is a broad term describing an approach to health policy and service provision that includes both services delivered to individuals (including patient pathways) and the general population. [19] Primary care (PC) refers to “family doctor-type” services delivered to individuals, whereas some frameworks [19, 20] use PC to assess PHC components.

**Fig. 1:**
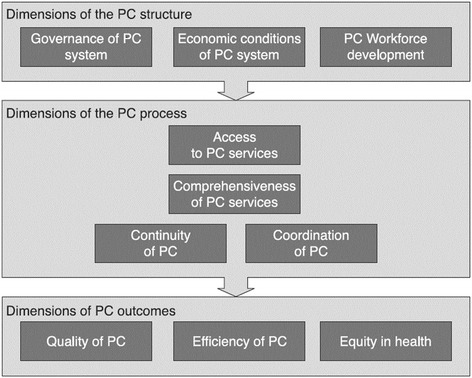
Adopted Primary Health Care System Framework (reproduced with permission) [20]

The adopted operational integration model for this study was based on the Donabedian approach, [21] combining the basic PHC principles presented by Starfield [22] and the chronic care model [23]. Following a systematic literature review, the dimensions of PHC as reported in the work of Kringos and colleagues, [24] were selected as the most appropriate for the Greek healthcare system. According to Kringos and colleagues, [20] PHC is viewed as a complex system comprising three levels: (a) structures, (b) processes, and (c) outcomes. [20] Each level is composed of dimensions encompassing a range of key attributes/features (Fig. 1).

### Setting and sample

The scope and range of PHC services in Greece tends to be broad, with services provided by entities that are not solely PHC oriented. For this project, the team adopted the PHC definition as established by legislation in effect at the time the study was designed (Laws 3235/2004, 3918/2011, and 4238/2014), classifying all of the following as PHC units:

- NHS Rural Health Centres, along with their satellite Clinics
- NHS Hospital Outpatient Clinics
- Outpatient Units within the National Health Service Organization (EOPYY)
- Private general practices affiliated with the EOPYY
- Municipal agencies (“Care at Home”, Senior Citizen Services, Outpatient Centres for Nursing Care of the Aged, Municipal Clinics)
- Other facilities (outpatient mental health units, rehabilitation units, ambulatory/community care units)

Simple random sampling was performed to identify a representative sample of PHC units according to type of provided services. Study setting included parts of the 2nd Health Region (HR) of Piraeus and Aegean and the 7th HR of Crete, with a total of 12 and 27 eligible units, respectively. Approximately 50% of all eligible PHC units per setting were included in the study (*N* = 22 PHC units, 7 units from the 2nd HR and 15 units from the 7th HR). Sample units were grouped under the following six categories according to provider setting:

1. Rural Health Centres/Satellite Clinics
2. Emergency Departments / National Centre for Emergency Assistance (EKAB)
3. Outpatient Clinics/Private practices/Diagnostic Centres
4. Community-based agencies for vulnerable or at-risk groups/Home Care Programs
5. Mental Health Clinics
6. Prevention and Rehabilitation Centres

For the purpose of this study, patient encounters were defined as any physical contact between a patient and a PHC practitioner, during which an assessment or clinical activity was performed. Eligible patient encounters were grouped by type of PHC service sought for into 4 categories: chronic disease care, urgent/acute episodic care, preventive care, and home care. This encounter grouping was based on the approach of Starfield, [22] and the observed utilisation patterns for the most frequently sought services in Greek PHC settings. One of the following inclusion criteria had to be fulfilled: a) in need of chronic care (registered patient with a regular follow-up appointment for a chronic condition, based on attending physician orders); b) in need of urgent or acute care (registered patient seeking unplanned episodic care); c) in need of prevention [registered patient with routine appointment for primary prevention (e.g. vaccination, smoking cessation, etc.) or secondary prevention (e.g. Prostate-specific antigen (PSA) measurement, Papanicolaou (Pap) test); d) in need of home care [chronic patient with disability (physical, psychological or mental), who was registered in the home care program and had been followed by a public PHC unit physician (excluding private unit practitioners)]. All patients under the age of 18 years or not speaking Greek fluently were excluded.

Given the nature of the study, the selected methods of analysis, and the modelling processes using computerised techniques, no power analysis was conducted. A minimum of 12 patients per unit was set as a prerequisite for unit inclusion (convenience sampling), with higher patient flow units contributing more patients and with no upper limit. Out of 305 eligible patients attending the units during the 3-week study period (August – September, 2015), a total of 282 patients were enrolled in the study (92.5% response rate).

### Tools and data collection process

A web-based platform entitled *“Information system for operational integration assessment within PHC units”* was developed to assess patient pathways, evaluate integration levels and propose improvement actions (http://ld.datacenter.uoc.gr/). This platform featured an online questionnaire consisting of four sub-scales, according to type of PHC service sought for, i.e., chronic disease care, urgent/acute episodic care, preventive care, and home care. Questions covered the following pathways: a) first contact, b) patient management/treatment, c) referral, and d) follow-up. Information regarding workflows, the role of each HCP, protocols/documents utilised and time required was collected. As a reminder, the definition of operational integration was clearly stated on the instruction page (first page), as well as on the lower part of each page of the questionnaire.

The questionnaires were completed by research associates (RAs) of the CSFM. The RAs used tablets to access the web-based platform. All RAs were extensively trained in theoretical and technical aspects of this project (i.e., integration conceptual framework, using the web-based platform, and resolving technical problems). Required qualifications for RAs included holding a health science degree, proven experience in health systems research, ability to use online platforms and having good communication skills. They visited the selected units for three weeks, identifying eligible patients at the reception (first contact). Upon establishing eligibility, RAs explained the purpose of the study in more detail. Upon receiving agreement for participation, they accompanied the patients, tracking pathway progress within the PHC unit and filling in the questionnaire directly into the web-based platform. At the end of data collection, one full-time HCP per unit was trained on the job to ensure continuous use of the platform and evaluation of the unit’s integration level. This maximised the impact of the study through a continuous quality improvement tool that was made available to the units.

### Analysis

A five-point Likert-type scale, i.e., 1 “minimal”, 2 “poor”, 3 “basic”, 4 “partial”, and 5 “operational”, coupled with an algorithm, was used to evaluate unit integration level. Based on the developed algorithm, different mathematical weights were used per PHC dimension in order to estimate the final scores. Weights ranged from 0.2 to 0.5 within each dimension’s characteristics, leading to a final scoring ranging from 1 to 5 points (cut off = 2.5). Furthermore, a minimum integrated unit was defined as one that scored from 1 to 1.5 (1 ≥ x ≥ 1.5), while a poorly integrated unit scored from 1.5 to 2.5 (1.5 > x ≥ 2.5). Scores ranging from 2.5 to 3.5(2.5 > x ≥ 3.5) points characterised a basically integrated unit and scores from 3.5 to 4.5 point (3.5 > x ≥ 4.5) indicated a partial integration. A well-integrated unit (operational integration) ranged from 4.5 to 5.0 (4.5 > x ≥ 5.0).

Evaluation was computed in a hierarchical manner, starting from the lower level framework features, going through dimension levels, and reaching the top level for overall integration score. In each layer, the weighted sum of the respective elements (i.e., questionnaire field, feature, and dimension) was computed according to the Simple Additive Weighting (SAW) method [25]. Weight calculation was performed according to the Analytic Hierarchy Process (AHP) through input from highly qualified experts. Feature measurability was enforced via an expert-driven assignment of relevant questionnaire items. The following set of equations summarises, in a hierarchical manner, the evaluation of overall integration level:

$$ {score}_i^{feat}=\frac{\sum_q{f}_q\left({value}_q\right)}{\left|{Q}_{feat}\right|}- \mathrm{Level}\ 1\ \left(\mathrm{Bottom}\right) $$

$$ {score}_i^{\mathrm{dim}}=\sum \limits_{feat}{w}_{feat}\cdot {score}_{feat}^i- \mathrm{Level}\ 2 $$

$$ {score}_i=\sum \limits_{\dim }{w}_{\mathrm{dim}}\cdot {score}_{\mathrm{dim}}^i- \mathrm{Level}\ 3\ \left(\mathrm{Top}\right) $$

Where ${score}_i^{feat}$ is the score of feature *feat* for unit *i*, *value*_*q*_is the value completed by unit professional for question *q* of the questionnaire where this question has been mapped to feature *feat* and belongs to the set |*Q*_*feat*_| of mapped questions of this feature, *f*_*q*_is the score function for question *q*, ${score}_i^{\mathrm{dim}}$is the score of dimension *dim* for unit *i*, *w*_*feat*_ is the weight of feature *feat*, *score*_*i*_is the overall score for unit *i* and *w*_dim_is the weight of dimension “dim”.

Ratings were exported for assessing operational integration in total for each unit, each type of patient, and each dimension and feature, respectively. Analysis was carried out via a stand-alone software, implemented in the Java programming language, which realised the Multi Criteria Decision Making (MCDM) method of quantitative process modelling. [26]

### Quantitative process Modelling

While a great number of quantitative process modelling methodologies has been suggested in the literature, MCDM was selected for the current study. This approach allows data collection, analysis and modelling of information collected by interdisciplinary teams or individuals with PHC expertise, as demonstrated by this project team (physicians, nurses, social workers, health administrators, and computer engineers). In addition, it does not require large sample sizes; input from two participants is deemed sufficient to proceed with modelling processes. According to MCDM, two sequential steps were followed:

- *First step*: modelling the four processes by monitoring current pathways, via the completed questionnaire parts, per type of PHC encounter, i.e., chronic conditions/diseases, urgent or acute problems/symptoms, prevention, and home care services. This was accomplished by: (a) collecting all pathway steps and mapping them to the four aforementioned processes, (b) matching steps with the same semantics, (c) abstracting multiple steps into an equivalent overall task or subprocess, (d) determining the control flow of the process (abstracted) tasks or subprocesses. Models of the four processes are available through the Additional files.
- *Second step*: development of optimal processes. An interdisciplinary project team utilised the first step intermediate product for modelling the four PHC processes/pathways. The goal was to formulate a set of well-prescribed steps for each process and map them to particular roles that characterise each PHC unit. To this end, each process should be extended and cover all possible paths, involving all possible respective steps, regardless of whether they were mandatory or optional, or whether they were frequently or rarely executed. This allowed examination and determination as to which of the proposed modelled steps or paths were actually executed in practice and how these differed from the ones being followed at that time.

The second step comprised three main phases: a) mapping of generalised tasks/subprocesses into a set of steps with a particular logic sequence; b) validation of the derived processes according to literature, team experience, and outcomes from previous relevant projects; c) development of the final model for each process through simulation and error detection algorithms, with special focus on compact and hierarchical product models of high quality.

The above steps were visualised through diagrammatic modelling of the generalised processes (workflows) in the Microsoft Visio tool. This resulted in a simplified visual modelling for each process, comprehensive but readily understandable by non-experts in process modelling, and, thus, facilitating communication among interdisciplinary team members. An enriched dictionary of common steps (e.g., history taking) was used, while each step was also linked to an abstract/generalised task (e.g., patient reception). The overall (quantitative) assessment of current integration levels, as well as per type of patient and dimension, was estimated using the SAW algorithm according to a similar hierarchical manner as in the case of the qualitative evaluation. Results were then produced and illustrated in pie and bar charts. During the final phase (step 2), the Microsoft Visio diagrams were transformed into standard process models (according to the Business Process Model and Notation, BPMN) and were tested and simulated via the ADONIS (http://en.adonis-community.com/) business process management tool (Additional files).

## Results

The overall integration of current processes varied among units, with the majority (57%) scoring 3, basic integration, in the Likert-type scale. In addition, 29% and 14% of the units presented poor and partial integration, respectively. None of the units scored at the highest and lowest ends (operational or minimal integration).

### Integration level by type of PHC services

**Fig. 2:**
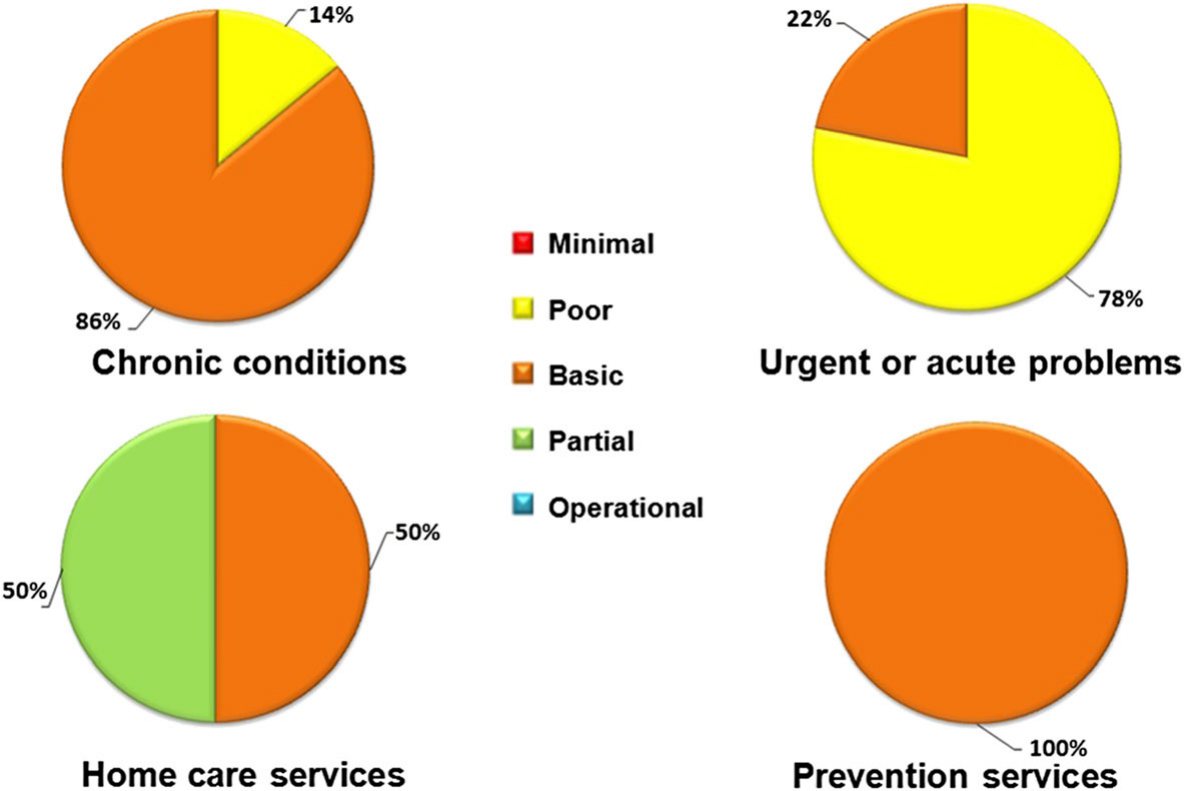
Integration level by type of PHC services offered

Figure 2 shows integration levels by type of PHC services sought for by patients. Units for patients seeking care for chronic conditions presented basic (86%) and poor (14%) integration. For units offering urgent or acute care, the majority (78%) scored poor integration and only 22% basic. Integration among home care services was evenly split between partial (50%) and basic (50%). Last, all units providing prevention services scored at the basic level (100%).

The current patient processes are presented in two selective diagrams in Additional file 1: Figure S1; Additional file 2: Figure S2, while optimal processes are extensively presented in the following section.

### Optimal processes by type of PHC services

- Chronic care management

The optimal workflow diagram for chronic care management is depicted in Additional file 3: Figure S3. This process comprises four subprocesses that are associated with: 1) patient reception/intake, 2) treatment, 3) referral, and 4) monitoring. These subprocesses are sequentially executed. Patient reception and intake are considered an independent step during which a non-physician HCP records patient demographics and medical history. Patient treatment is a subprocess that begins with assignment to a professional on the premise of a scheduled appointment. A scheduled appointment may involve more than one visit to different PHC professionals. Internal referrals can be made to either physicians or non-physician HCPs. A non-physician HCP can refer to a physician, while a physician can refer to a non-physician HCP or another physician of a different specialty, within the unit.

Patient treatment provided by an HCP involves the actual treatment, as well as consultation, including update of the health record. Actual treatment may include: behavioural change (lifestyle pattern) consultation, self-care management and training, medication prescription or psychological support. Treatment by a physician includes: update of patient health record and actual treatment, such as behavioural change (lifestyle pattern) consultation, self-care management and training, medication prescription, ordering laboratory or diagnostic tests. The physician can perform internal or external referrals to other physicians and other HCPs.

External referral is performed upon writing a referral note. The patient can be transferred either by own means or via the National Centre for Emergency Assistance (EKAB). External referral to a physician of a different specialty may be made to public and private PHC or secondary health care (SHC) units. Public PHC units include: the National Primary Healthcare Network (PEDY), mental health clinics, regular outpatient clinics or satellite clinics. Private PHC units include either private practices or private diagnostic centres. Public SHC units are regular outpatient clinics, emergency departments or hospital laboratories, while private SHC units can only be a private clinic.

External referral to an HCP involves either the public sector affiliated with PEDY, mental health clinics or satellite clinics or the private sector affiliated with private practices.

The last subprocess, patient progress monitoring, comprises three sequential steps: a) assessment of disease/symptom management, b) monitoring, and c) patient and family briefing/feedback.

b) Urgent or acute episodic care management

Optimal urgent or acute care management process is presented in Additional file 4: Figure S4**.** Overall, it follows the same pattern as the optimal chronic disease management process with main differences in treatment and referral making.

A nurse or a physician can administer patient treatment. Patient treatment by a nurse includes one or more of the following steps: a) medical history recording & triage, b) first aid or c) first aid provision, medication and/or psychological support. Patient treatment by physician involves: a) medical history recording & triage, b) first aid and medication treatment or c) first aid, medication treatment and laboratory or diagnostic exams.

Upon treatment, the nurse can only make an internal referral to a physician, while a physician can conduct both internal and external referrals to physicians of a different specialty or to non-physician HCPs. In case of an external referral, the physician should be affiliated either to the Primary National Health Network, Mental Health units, Outpatient Clinics or to Health Centres. On the other hand, an external HCP who receives a referral can be affiliated to the Primary National Health Network or the Mental Health units.

c) Preventive care management

The optimal prevention process (Additional file 5: Figure S5) is similar to the previous processes with patient reception/intake and monitoring of health outcome being the same. Incident treatment follows the same pattern with the main difference being the actual treatment.

Treatment by a non-physician HCP can involve primary or secondary prevention procedures. Treatment by a physician can include secondary prevention procedures, medication prescription, or ordering laboratory or diagnostic exams. The physician has the authority to conduct internal or external referral to both non-physician HCPs and other physicians. For external referrals, writing a referral note is necessary. Referral to an HCP can be made within the Primary National Health Network, mental health clinics, rehabilitation centres or private clinics. When referring to another specialty physician, an affiliation with outpatient clinics, private clinics or private diagnostic centres is required.

d) Home care management

The optimal home care process model is depicted in Additional file 6: Figure S6. This process follows a different pattern due to the uniqueness of the setting, a patient’s home rather than a formal PHC unit environment.

This process starts with two subprocesses, performed by nursing and social care professionals, which can be executed sequentially or interchangeably. The first step involves patient needs assessment, followed by one or more of the next steps: a) planning necessary care/interventions, b) implementation of care/interventions, and c) evaluation of care/interventions. Health needs assessment includes biological/physical as well as socio-economic and mental needs.

Care and intervention planning includes the conditional performance of four sub-steps, from the simplest to the most complex: a) lifestyle change, b) lifestyle change and self-management/training regarding medication regime, c) lifestyle change, self-management/training of medication regime and mental support, and d) lifestyle change, self-management/training of medication regime, mental support and laboratory tests.

Implementation of care and intervention includes: a) a personalised care plan, b) identifying resources, and c) connecting with community resources.

After each subprocess ends, a referral can be made to the patient’s family physician. Such referral can involve a home visit by the family doctor or a patient visit to the PHC unit where the attending physician works. In the first case, once the physician visits the patient, he/she can be referred to a PHC unit for further examination or assessment. Referral can be made to the Outpatient Clinics, the Emergency Department or the Hospital Diagnostic Laboratories.

Finally, program or care evaluation includes one or more of the following sub-steps: a) patient/caregiver briefing, b) patient/caregiver training, and c) re-evaluation of care/intervention.

## Discussion

### Main findings

This study met its objectives of assessing patient pathways within PHC units and proposing optimal integration processes. Integration per type of PHC service was measured for the first time in Greece and was found to greatly vary from poor to basic levels. Main limitations to achieving operational integration included: a) lack of an IT system that could support referral, patient history/EHR and prescribing within and across units, and most importantly across levels of care, b) absence of gatekeeping, c) incomplete or missing patient lists to facilitate monitoring, referral and prescribing patterns at practice/unit level and at district level, and d) absence of standardised patient pathways to facilitate virtual path and movement. To this end, optimal processes were developed in the form of diagrams that could facilitate evaluation of current process within the context of Greek PHC units. Members of the interdisciplinary research team developed detailed, comprehensive processes that covered different typical cases of patients within all possible PHC unit types and settings. These processes could be implemented by an execution runtime system to enable operational integration, tracking and overall monitoring. This runtime system is expected to enable modification of each process, as well as the dynamic management of computerised and human resources, according to each unit needs. It could also enable real-time analysis of stored data to assess levels of integration and provide optimisation guidelines.

### Discussion in view of the literature

There is growing consensus that Greece should work towards operational integration by allocating resources in a cost-effective and quality-assured manner [27]. This is considered a challenging task due to the lack of data on current integration levels and the requirements of PHC units [28]. In addition, key performance indicators for processes should be developed and adopted in order to establish sustainable integrated care models [29]. The present study attempted to map the current integration levels and processes within the Greek PHC units, as well as to develop the optimum processes that should guide the national operational integration model.

Interestingly, despite the widespread budgetary and human resource problems, integration among home care provider units scored at higher levels. A recent SWOT analysis of home healthcare service operations identified a lack of an integrated institutional framework as a major deficit [30]. The pivotal role of nursing in case managing home care recipients and improving quality of life can be instrumental in integrating services and achieving seamless care [31, 32]. Yet, policy makers in Greece have failed to recognise existing evidence. Given the reported high level of operational integration, this study provides further support in the direction of expanding home care services led by nurses or social workers. Viewed as an action call to health policy makers, as well as healthcare institutions and professional organizations, to cover a larger proportion of the urban population in need of community-based and home-based skilled nursing care, the study tools can provide evidence and further guidance in that direction.

Existing literature has revealed a wide range of parameters that contribute to poor integration, including absence of an interoperable IT system with standardised flows, unequal distribution of equipment, staff and other resources [31–33]. Our study supports previous findings indicating that the lack of standardised processes and evidence-based guidelines widens the gap between theory and practice among PHC units.[7] Therefore, there is a negative impact on continuity, coordination, comprehensiveness and quality of PHC services for all patient categories [15, 17]. A systematic review by Van der Klauw and colleagues (2014) suggested that effective IT systems constitute a core element for operational integration within PHC [34]. This promotes patient-centeredness and facilitates communication between healthcare professionals and patients (e.g., follow-up, patient training and active self-management) [13, 28].

Reform of the Greek healthcare system during this intense austerity period, should be guided by best practices from other countries, adjusted according to current findings [35]. The theoretical model by Kringos and colleagues [20] coupled with the Chronic Care Model is strongly recommended by the authors and other researchers [36, 37]. Greece could become a case study for highly burdened healthcare systems aiming to streamline operations and achieve sustainability.

### Strengths and limitations

To our knowledge, this is the first study to capture the level of integration within PHC units by measuring specific indicators. The high response rate (92.5%) and the relatively large and representative sample strengthen the generalisability of our findings. The developed online tool could be utilised by both PHC units and the Ministry of Health to systematically monitor integration and take adequate steps towards reform and quality improvement. Another unique component is the project’s interdisciplinary team that conceptualised the study design, mapped and interpreted data, and designed optimal processes. This is in line with experience from the UK that supports involvement of healthcare providers in the design of new operational integration models [38, 39].

Study limitations include the utilisation of a concrete operational definition of integration, which may not satisfy other definitions used in the literature. Participating PHC professionals and patients were not asked about their perception of integration, as this was not part of the study aim. Assessment of integration was based on the measurable indicators of our theoretical framework. This information was captured in real-time mode and based on the actual patient workflows within the PHC unit. Actual integration of patient flows within PHC units was monitored, but we did not assess patient perceptions regarding integration. Furthermore, assessment of operational integration was performed in only two health regions; therefore, results may differ at the national level. However, every effort was made to secure representation of all six types of PHC units, based on type of services offered. There is also potential for information bias due to the self-administered nature of the questionnaire, which might have resulted in an overestimation of unit integration level. Last, due to major structural changes in the healthcare sector at the time this study was carried out, mapping of PHC units was quite challenging, requiring frequent methodological adjustments.

### Implications

Immediate actions towards patient-centred care are necessary in order to operationally integrate all provided services and existing functions of the PHC system. Health policymakers should adopt an evidence-based action plan that ensures and safeguards patient-centredness, comprehensiveness, sound coordination, and continuity. Linking the developed web-based platform with the existing healthcare information system is required in order to systematically evaluate efficiency (services, procedures, resources, manpower, and outputs). This could strengthen efforts to address new challenges such as poverty, an aging population, increasing healthcare expenditures, reduction of resources, rapidly changing epidemiological trends indicating mental disorders, and cardiovascular disease as leading causes of morbidity.

## Conclusions

This national study revealed average or below average levels of patient-level integration within PHC units, with variations based on type of PHC services rendered. Indications for a fragmented and ineffective healthcare system in need of reform were evident, particularly when assessing the existing patient care pathways. Towards that end, this study generated new evidence from Greece that could offer valuable insights to other Southern European countries with similar characteristics. The web-based evaluation tool, along with the proposed patient-level operational integration model, could become the core elements for an overall sound and cost-effective primary healthcare system, a system where professionals, along with patients, are motivated and empowered to work collectively towards integrated patient-centred care.

## Additional files

Additional file 1: Figure S1. Current process workflows for managing patients with urgent or acute symptoms within PHC units. Illustrates the actual patient flows within the PHC units, as they were currently monitored and mapped by the project. It focuses on patients with urgent or acute symptoms seeking for PHC services. (JPEG 257 kb)

Additional file 2: Figure S2. Current process workflows for managing patients with chronic conditions within PHC units. Illustrates the actual patient flows within the PHC units, as they were currently monitored and mapped by the project. It focuses on patients with chronic conditions seeking for PHC services. (JPEG 77 kb)

Additional file 3: Figure S3. Optimal processes workflow for patients with chronic disease. Distributed the optimal patient flows within the PHC units, as they are proposed by the project. It depicts the processes workflows of patients with chronic conditions seeking for PHC services. (JPEG 150 kb)

Additional file 4: Figure S4. Optimal processes workflows for patients with urgent or acute problems/symptoms. Distributed the optimal patient flows within the PHC units, as they are proposed by the project. It depicts the processes workflows of patients with urgent or acute problems/symptoms seeking for PHC services. (JPEG 182 kb)

Additional file 5: Figure S5. Optimal processes workflows for patients in need of prevention services. Distributed the optimal patient flows within the PHC units, as they are proposed by the project. It depicts the processes workflows of patients seeking for prevention services in PHC units. (JPEG 5066 kb)

Additional file 6: Figure S6. Optimal processes workflows for patients in need of home care services. Distributed the optimal patient flows within the PHC units (i.e. home care), as they are proposed by the project. It depicts the processes workflows of patients in need of home care service. (JPEG 4616 kb)

## Abbreviations

BPMN: Business Process Model and Notation
HCP: Healthcare Professional
IT: Information Technology
MCDM: Multi Criteria Decision Making
NHS: National Health System
NSFR: National Strategic Reference Framework
PHC: Primary Health Care
SAW: Simple Additive Weighting
SHC: Secondary Health Care
WHO: World Health Organization
