# Supplementary figures and images for: Operational integration in primary health care: patient encounters and workflows
Source: BMC Health Serv Res. 2017 Nov 29;17:788. doi: 10.1186/s12913-017-2702-5 (PMC5706391; doi:10.1186/s12913-017-2702-5)

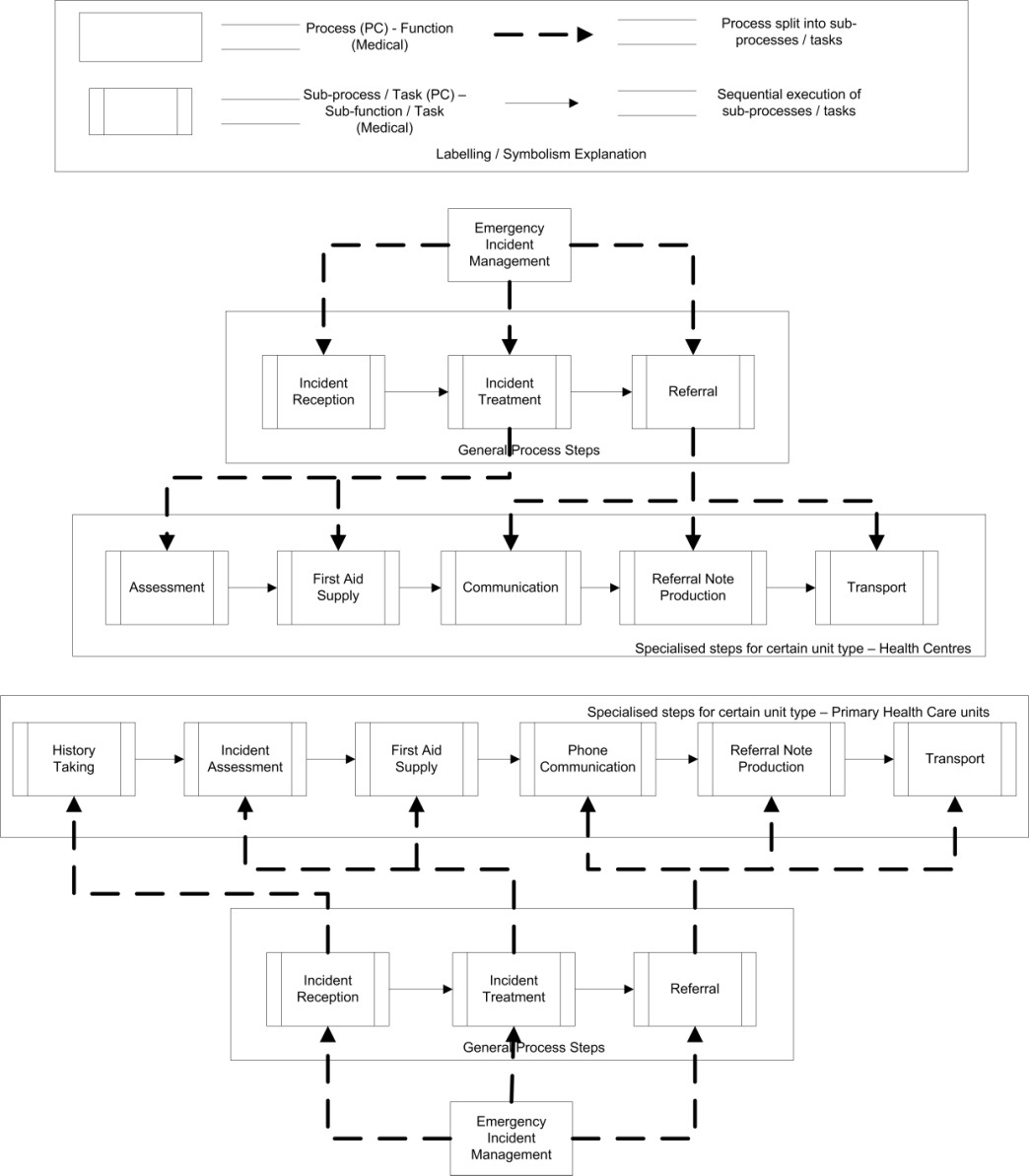

Supplement: Supplementary file 1 — Current process workflows for managing patients with urgent or acute symptoms within PHC units. Illustrates the actual patient flows within the PHC units, as they were currently monitored and mapped by the project. It focuses on patients with urgent or acute symptoms seeking for PHC services. (JPEG 257 kb) [file 12913_2017_2702_MOESM1_ESM.jpg]

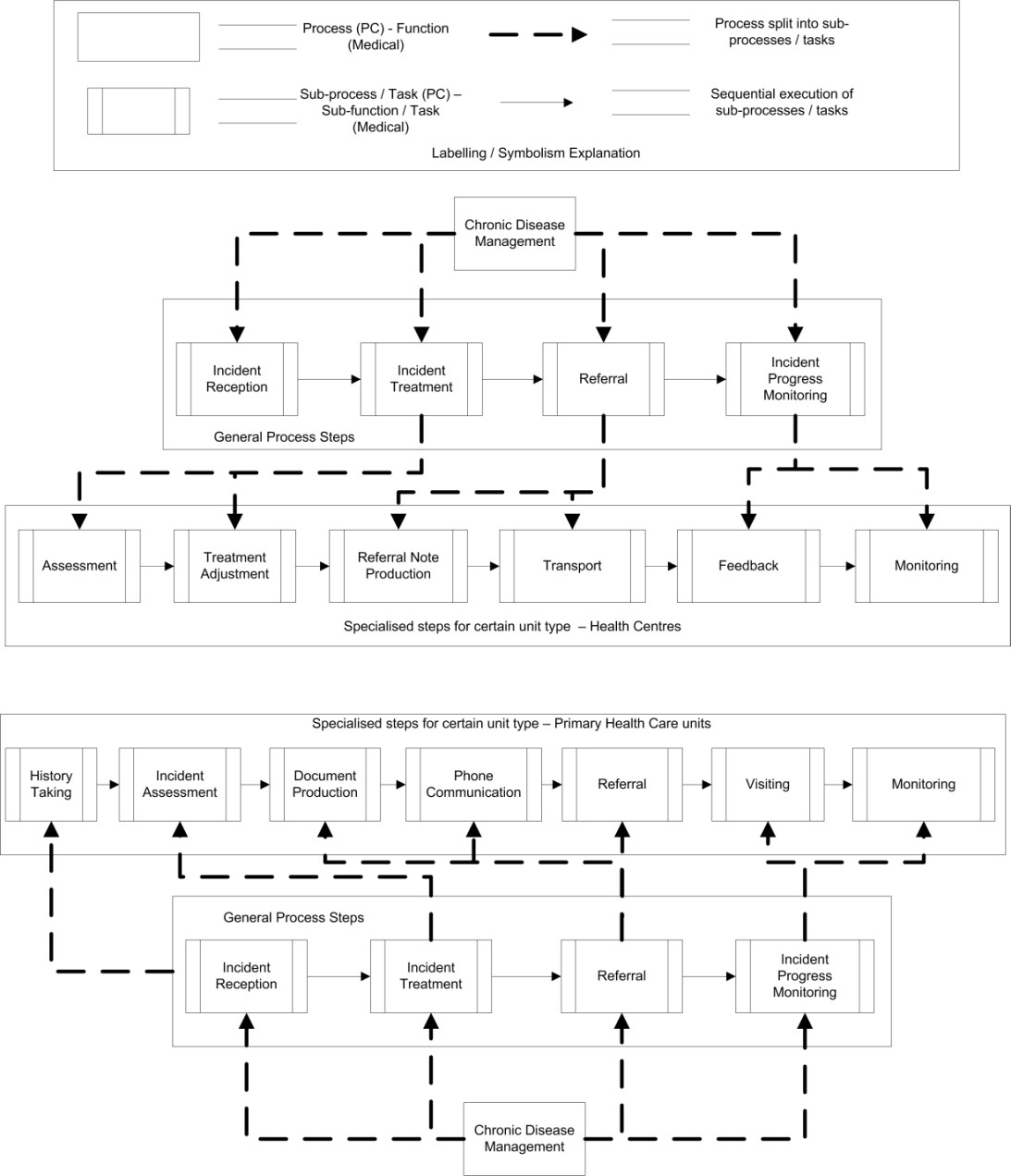

Supplement: Supplementary file 2 — Current process workflows for managing patients with chronic conditions within PHC units. Illustrates the actual patient flows within the PHC units, as they were currently monitored and mapped by the project. It focuses on patients with chronic conditions seeking for PHC services. (JPEG 77 kb) [file 12913_2017_2702_MOESM2_ESM.jpg]

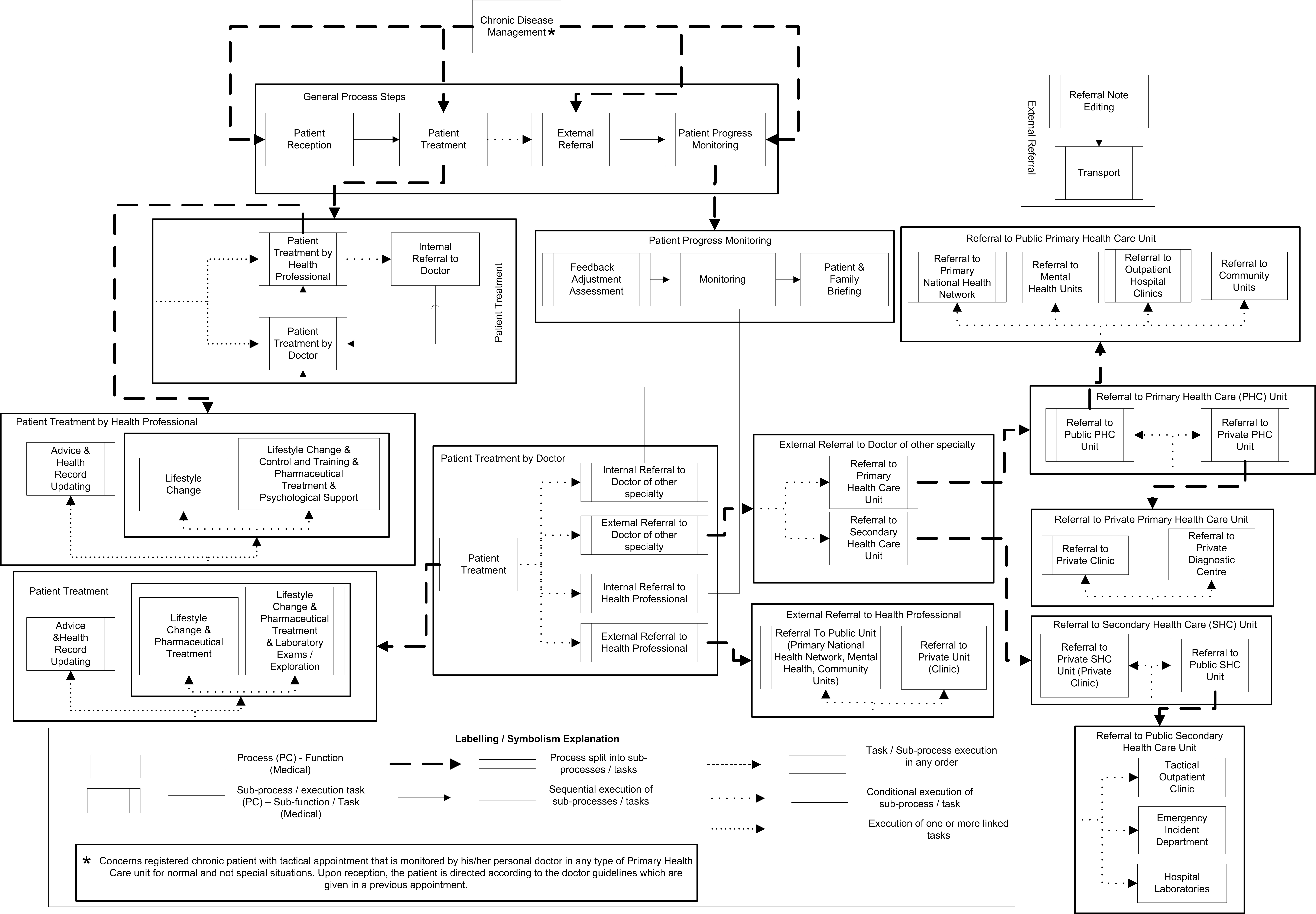

Supplement: Supplementary file 3 — Optimal processes workflow for patients with chronic disease. Distributed the optimal patient flows within the PHC units, as they are proposed by the project. It depicts the processes workflows of patients with chronic conditions seeking for PHC services. (JPEG 150 kb) [file 12913_2017_2702_MOESM3_ESM.jpg]

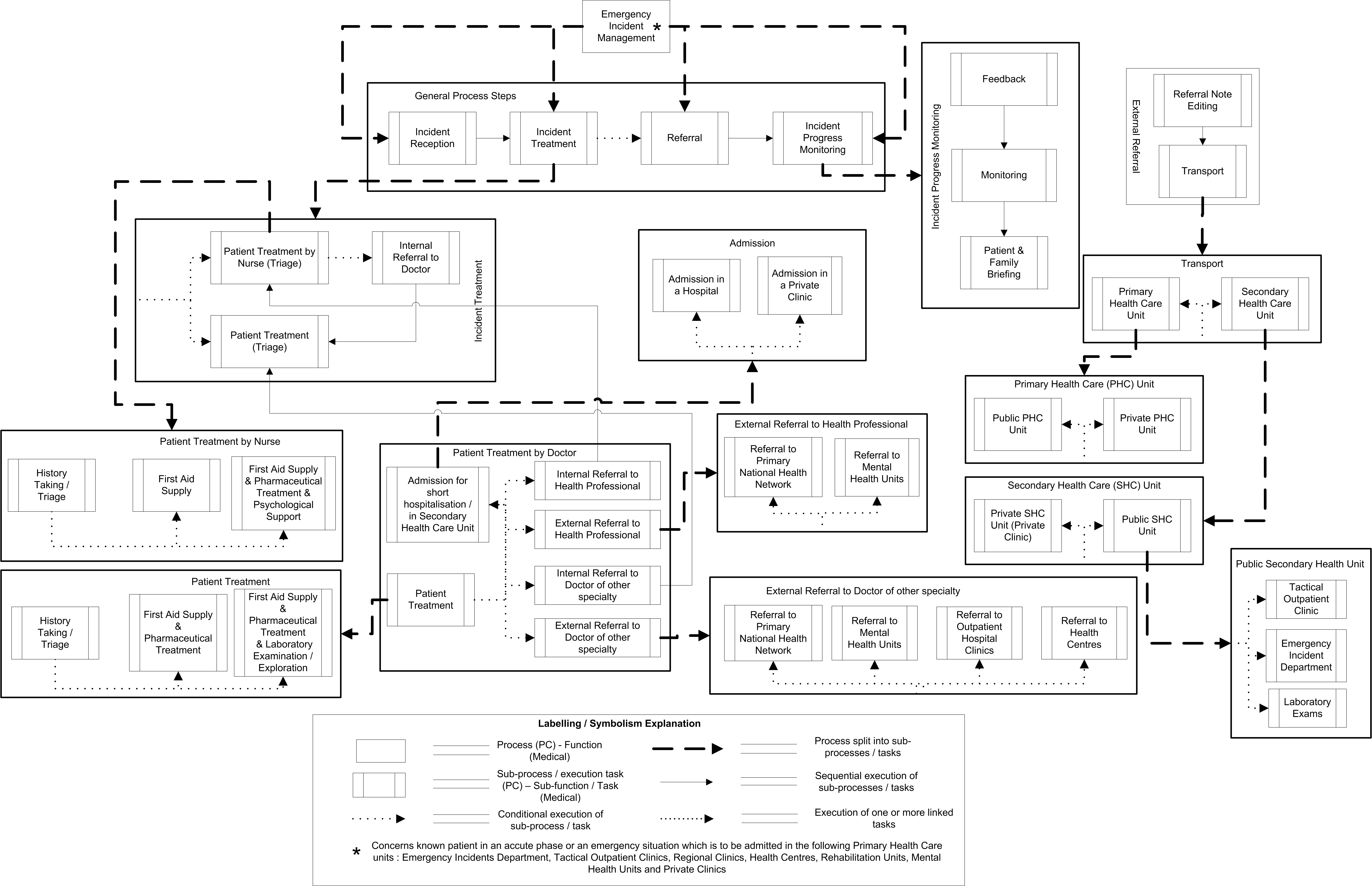

Supplement: Supplementary file 4 — Optimal processes workflows for patients with urgent or acute problems/symptoms. Distributed the optimal patient flows within the PHC units, as they are proposed by the project. It depicts the processes workflows of patients with urgent or acute problems/symptoms seeking for PHC services. (JPEG 182 kb) [file 12913_2017_2702_MOESM4_ESM.jpg]

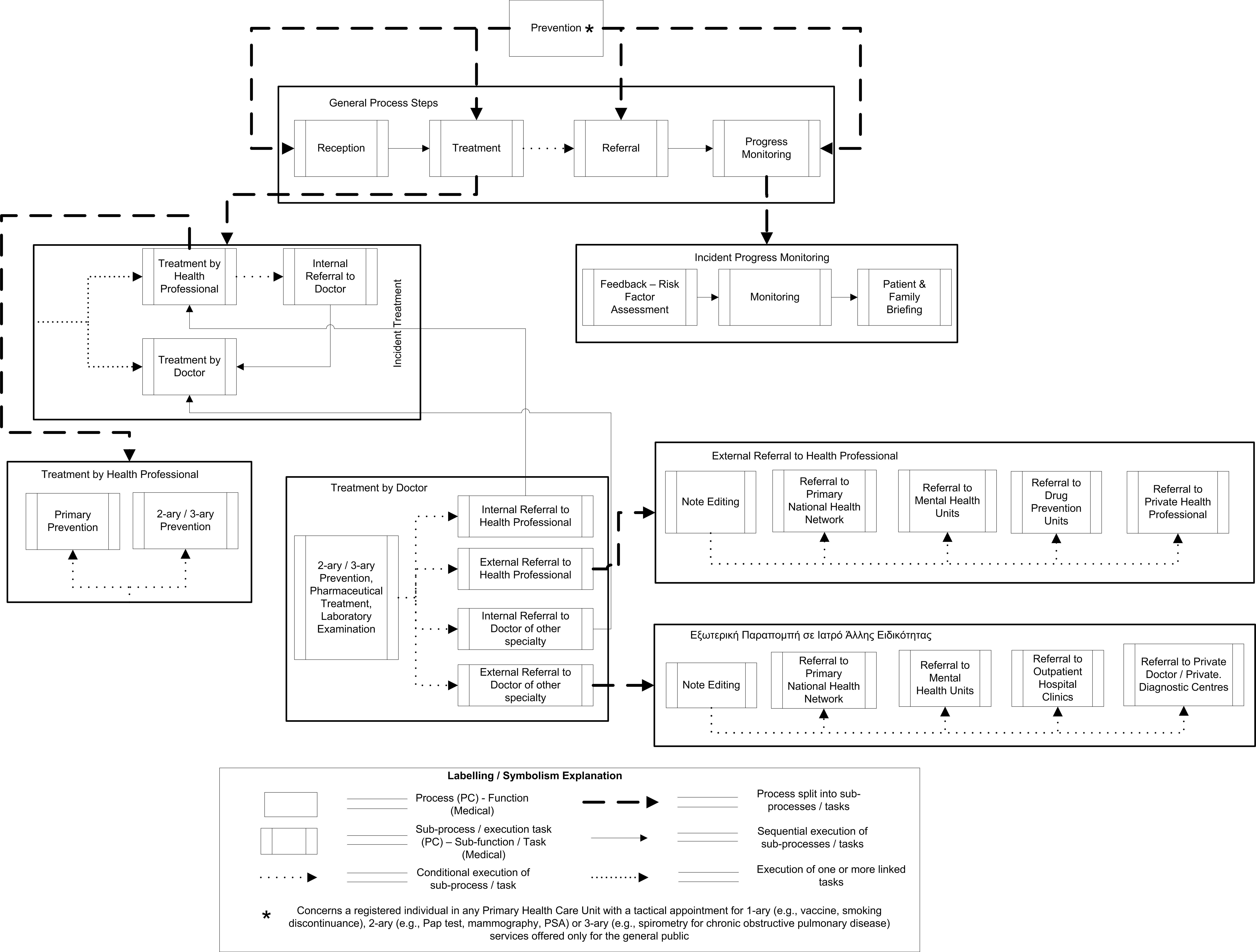

Supplement: Supplementary file 5 — Optimal processes workflows for patients in need of prevention services. Distributed the optimal patient flows within the PHC units, as they are proposed by the project. It depicts the processes workflows of patients seeking for prevention services in PHC units. (JPEG 5066 kb) [file 12913_2017_2702_MOESM5_ESM.jpg]

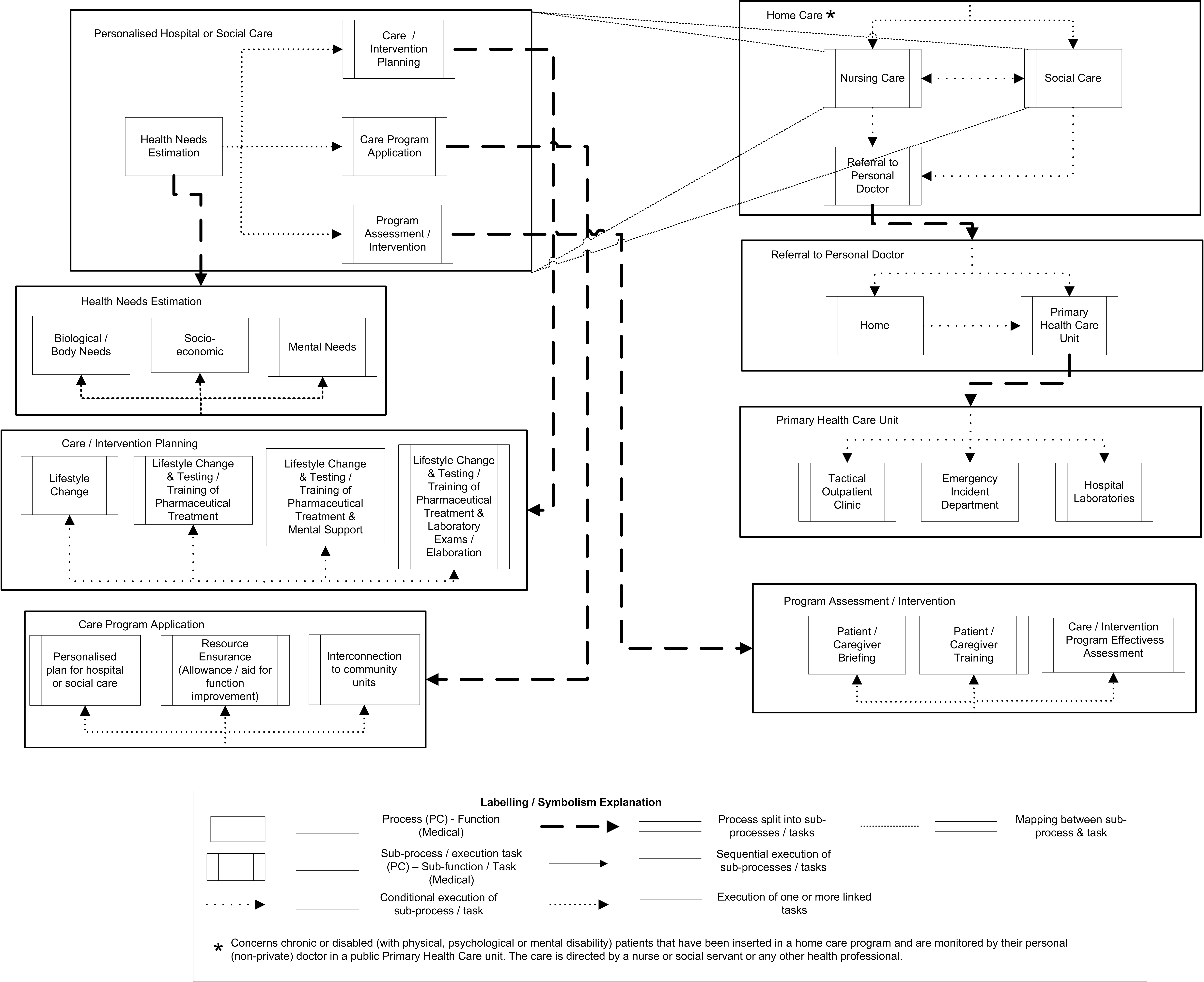

Supplement: Supplementary file 6 — Optimal processes workflows for patients in need of home care services. Distributed the optimal patient flows within the PHC units (i.e. home care), as they are proposed by the project. It depicts the processes workflows of patients in need of home care service. (JPEG 4616 kb) [file 12913_2017_2702_MOESM6_ESM.jpg]
